# The Neurobiology of Pathological Fatigue: New Models, New Questions

**DOI:** 10.1177/1073858420985447

**Published:** 2021-01-15

**Authors:** Annapoorna Kuppuswamy

**Affiliations:** 1 Department of Clinical and Movement Neuroscience, Institute of Neurology, University College London, London, UK

**Keywords:** agency, exteroception, fatigue, interoception, multiple sclerosis, Parkinson’s, sensory attenuation, stroke

## Abstract

The last decade has seen the emergence of new theoretical frameworks to explain pathological fatigue, a much neglected, yet highly significant symptom across a wide range of diseases. While the new models of fatigue provide new hypotheses to test, they also raise a number of questions. The primary purpose of this essay is to examine the predictions of three recently proposed models of fatigue, the overlap and differences between them, and the evidence from diseases that may lend support to the models of fatigue. I also present expansions for the sensory attenuation model of fatigue. Further questions examined here are the following: What are the neural substrates of fatigue? How can sensory attenuation, which underpins agency also explain fatigue? Are fatigue and agency related?

We hence perceive a primary law of fatigue and of sensation, namely that their intensity is not at all proportional to the intensity of the external cause that produces them.

—Angelo Mosso, La Fatica, 1891

The notion that a stimulus for fatigue is subject to the laws of perception, that is, the stimulus can be amplified or attenuated, is an important observation. It implies a role for the brain, an organ of perception, in fatigue. More than a hundred years on, we acknowledge the role of brain in fatigue; however, how the brain generates an experience of fatigue in the apparent absence of a stimulus, as seen in pathological fatigue, is far from clear.

## Fatigue, the Cinderella of Affective Symptoms

Chronic, irreversible fatigue is a distressing symptom in several neurological conditions (Chaudhuri and Behan 2004; Penner and Paul 2017), yet until recently very little was known about what might cause such fatigue. A key reason being fatigue co-occurs with a wide variety of other affective symptoms and consequently thought to be a secondary symptom. Failure of treatments targeting the supposed primary problem to reduce fatigue lead to the gradual realization that fatigue is a primary problem driven by partially independent mechanisms. The strongest evidence for fatigue being independent comes from the lack of effect of antidepressants on fatigue, with some making fatigue worse. Moreover, almost everyone who suffers from depression, chronic pain, or sleep disturbances report fatigue, but a significant number with fatigue do not present with other problems. This raises a very important question that impacts on how we investigate and treat fatigue—Is fatigue a single construct? Is there more than one form of fatigue? Although fatigue is multidimensional, possibly requiring multiple strategies to combat it, the notion of a common mechanism underpinned by dysfunction in a fundamental property of brain processing is nevertheless conceivable. This is best captured by the definition proposed by Chaudhuri and Behan (2004), who describe fatigue as “a feeling arising from difficulty in initiation of or sustaining voluntary effort.” Here the focus is on voluntary effort without reference to domain specificity, therefore, be it fatigue triggered by physical or cognitive effort, effort is the common denominator, thereby any changes in effort related processing will result in fatigue. Although a common mechanism may drive fatigue, any application of common frameworks for purposes of intervention must take into account the multidimensional nature of fatigue as captured by fatigue questionnaires measuring physical, cognitive, and psychosocial dimensions of fatigue (Hewlett and others 2011), with a detailed discussion of the many dimensions of fatigue found elsewhere (Christensen and others 2008; Flinn and Stube 2010; Hagelin and others 2009; Joyner 2016; Whitehead and others 2016).

Several new models of pathological fatigue have emerged in the last decade—the sensory attenuation model of fatigue (SAF; Kuppuswamy 2017), the metacognitive theory of dyshomeostasis and fatigue (Stephan and others 2016) and inhibitory sensitization model of fatigue (Tanaka and others 2013). These new theoretical frameworks of fatigue are not completely new concepts, but existing principles of brain functioning have been repurposed to explain the emergence of fatigue. With this arises the questions—Can we then identify neural substrates of fatigue as predicted by these models? Do we have corroborating evidence from diseases in support of the new models of fatigue? This essay address these questions, after laying out the fundamentals of the three frameworks, discussing the convergences and divergences of the models and presenting an expansion of the SAF framework. Repurposing principles of brain function to explain fatigue also gives rise to another set of questions. What is the relationship between the behavior/experience which the principles were originally developed to explain, and fatigue? Sensory attenuation is a key phenomenon that underlies the sense of agency; therefore, how are fatigue and sense of agency related? I discuss the implications of a common driving principle and conclude with new directions of research for mechanistic understanding of fatigue.

## Models of Pathological Fatigue

Dyshomeostasis is the presumed cause of fatigue, both in health and disease. During exercise, increase in respiratory and heart rate increases metabolic demand, rising the core body temperature, drawing into action the temperature regulation systems(Ament and Verkerke 2009). With continued exercise, peripheral and central changes (Taylor and others 2016) lead to high perceived effort, task failure, and fatigue when homeostatic balance can no longer be maintained. In disease, the primary trigger is inflammation setting into motion a cascade of molecular and cellular events in the brain and the periphery. Effects of inflammation on dopaminergic (Felger and Miller 2012) and glutamate transport systems (Dantzer and others 2014; Haroon and others 2017; Rönnbäck and Hansson 2004) result in sickness behavior of loss of appetite, fatigue/anergia, and social withdrawal. Such behaviors are acute and reversible with removal of trigger. In some diseases which present with chronic inflammation, the continued presence of inflammatory cytokines may explain fatigue in the long term. But in some other diseases where prolonged inflammation is present such as multiple sclerosis (MS), there is no clear relationship between fatigue and inflammation (Chalah and Ayache 2018; Patejdl and others 2016). In diseases such as stroke and traumatic brain injury, where there is little long-term inflammation, fatigue is a significant chronic problem and although inflammation is predictive of early fatigue, is unrelated to long-term fatigue (Ormstad and others 2011; Shetty and others 2019; Su and others 2014; Wu and others 2015). To explain such prolonged fatigue, functional neural network dysfunction has been invoked, with three recent models being proposed.

The SAF framework (De Doncker and others 2018; Kuppuswamy 2017; Kuppuswamy and others 2015b) proposes that heightened effort perception, driven by poor motor related sensory gating underlies fatigue. The metacognitive theory of dyshomeostasis (Stephan and others 2016) is based on the principles of predictive processing. Here, greater attention afforded to interoceptive input due to repeated unfulfilled predictions, results in a reduction in allostatic self-efficacy, with poor allostatic self-efficacy being the basis of fatigue. The central sensitization model proposes that excessive activation of excitatory systems result in sensitization of the inhibitory systems resulting in a constant alarm signal indicating the need to rest and thereby an experience of fatigue (Tanaka and others 2013). I first present the predictions of the SAF framework and its implications for motor, visual, and auditory processing, followed by a comparison of the 3 models.

**Table:** 

Terminology	Definition
Sensory attenuation	A property of the brain that allows for distinction between self and externally generated stimuli by attenuating the sensory consequences of self-generated motor commands.
Homeostasis	A state wherein an organism is capable of maintaining a stable internal environment in the face of changing external environment.
Interoception	A sense of the internal state of the body, specifically organs that are normally not under volitional control such as the heart, lungs and gut.
Self-efficacy	Belief in ability to fulfil predictions (I can do that), this includes both nonconscious (those held by the brain) and conscious beliefs.
Sense of agency	A sense of control over consequences of an action (I did do that).

### Sensory Attenuation and Motor Effort Perception

When one moves, one does not explicitly experience the many brain computations involved in smooth execution of movement. But what is consciously perceived is a sense of effort assigned to a muscular contraction. Several elegant studies have shown that perceived effort can be altered by manipulating either afferent sensory input from the muscles (Bridgeman 2005; Brooks and others 2013; Gandevia 1982; Lafargue and others 2003; Luu and others 2011; Sanes and Shadmehr 1995), or disrupting sensory predictions (efferent; de Morree and others 2012; Slobounov and others 2004; Takarada and others 2014; Zénon and others 2015), which leads us to conclude that perceived effort (in a muscular contraction, the fundamental requirement for a physical action) is the psychophysical output of the process that integrates afferent input and sensory predictions. Sensory attenuation, a process wherein predicted sensory input is attenuated, and is implicated in motor control, has thus been proposed to underpin perceived effort. The SAF proposes that the gain of movement-induced sensory prediction errors is the basis of effort perception and high gain, or, poor sensory suppression explains high effort perception, a primary experience of fatigue (Kuppuswamy 2017). We recently showed that trait fatigue, but not state fatigue, positively correlated with perceived effort in low-force isometric grip, but not in the high-force conditions (De Doncker and others 2020b). The correlation with trait but not state fatigue suggests that fatigue on the day of testing was not driving the report of high effort. Moreover, a lack of correlation in higher force levels further substantiates SAF, as sensory attenuation holds true only in low force contractions. In MS, M1–S1 connectivity is compromised during an isometric grip task (Dell’Acqua and others 2010; Tecchio and others 2008), with such compromise being reflected in fatigue levels rather than movement execution parameters. Moreover, those with greater fatigue also showed greater increase in cortico-muscular coherence (synchronization frequency) in a fatigability paradigm, possibly reflecting increasing gain of prediction errors as muscle fatigue sets in Tomasevic and others (2013). Such greater increase could be driven by compromised M1–S1 connectivity seen in high-fatigue patients. Evidence thus far strongly favors the SAF framework to explain greater motor perceived effort in fatigue.

### Sensory Attenuation and Visual Effort Perception

**Figure 1.**
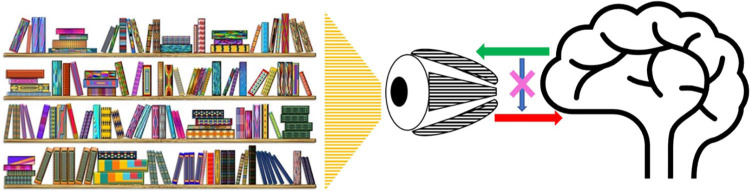
An illustration of the predictions of SAF (sensory attenuation model of fatigue) in visual processing: When viewing an image the eye scans the image by making several quick movements (saccades) to different parts of the image. As these eye movements are predicted (green arrow), the incoming sensory information (red arrow) during movement is suppressed, eliminating movement induced blurring of image and resulting in a stable image, known as saccadic suppression. SAF predicts that such movement induced suppression of visual information is lost (pink cross) resulting in visual processing becoming a high effort activity, eventually resulting in fatigue.

Unlike motor tasks where muscular activation per se entails experience of effort, activation of the visual end organs generally does not require much effort, with effort in visual tasks associated with attentional demands or task complexity. In fatigue, simple visual tasks are effortful and tiring, even in the absence of attentional demands and any obvious deficits such as ptosis or hemianopia. SAF predicts that greater effort perception in simple visual tasks is likely a psychophysical output of altered oculomotor control rather than deficits in high-order cognitive function. Oculomotor activation gives rise to motor corollaries inducing a suppression of visual input leading to a stable image despite movement of the eyes. This phenomenon of saccadic suppression is robust and a very well-studied phenomenon (Brooks and Cullen 2019). In SAF, the gain of movement-induced sensory prediction errors is the basis of effort perception and high gain, or, poor sensory suppression explains high effort perception (Kuppuswamy 2017). In this view, a poor eye movement–induced suppression of visual input may result in high perceived effort. In visual fixation, micro-saccades prevent decay of retinal image (Engbert 2006) and lack of sensory suppression during micro-saccades may also contribute to high-effort perception. Large, micro, and other kinds of saccades such as voluntary, reflexive, anti-saccades are all susceptible to disease processes (Willard and Lueck 2014) and significant oculomotor disturbances are seen in major neurological diseases where fatigue is a symptom such as MS (Ferreira and others 2017; Finke and others 2012), stroke (Dong and others 2013), and Parkinson’s disease (Helmchen and others 2012; Linder and others 2012). Reduced peak velocity, increased latency, and smaller amplitudes of saccades is seen in MS fatigue (Ferreira and others 2017; Finke and others 2012) and is thought to be sensitive markers of fatigue. In stroke, patients with no hemianopia or gaze palsy showed abnormal saccade parameters that did not correlate with motor, sensory, or cognitive dysfunction (Dong and others 2013), perhaps it is a marker of fatigue? Similarly, in Parkinson’s disease abnormal saccades do not track disease severity, progression, rigidity, tremor, or bradykinesia (Helmchen and others 2012; Linder and others 2012). Evidence strongly suggests saccadic abnormalities relate to fatigue, and qualitative studies show that greater visual effort (self-report) is a significant feature of fatigue (Barbour and Mead 2012; Whitehead and others 2016). In a sample of 117 chronic stroke survivors, about 40% reported visual abnormalities that were not a clinically diagnosed visual deficit, but related to how vision had changed poststroke, mostly with reference to perceiving visual stimuli. Interestingly, the average fatigue levels of those with self-reported visual perceptual disturbances was significantly higher than those without (unpublished observations from our stroke database cohort). Other quantitative evidence comes from studies that investigate subjective cognitive impairments, where cognitive impairments include information processing, executive functioning, and memory. As a large part of cognitive processing includes visual processing, the subjective measure could partly be a measure of visual effort. In stroke, there appears to be a relationship between negative affect and subjective cognitive impairment; however, no relationship with fatigue (Lamb and others 2013). In MS, presence of fatigue may influence subjective cognitive impairment scores such that the link between subjective and objective measures of cognitive impairment are severed (Hughes and others 2019). Interactions between visual effort and fatigue needs to systematically investigated to test the predictions of SAF framework (Fig. 1).

### Sensory Attenuation and Auditory Effort Perception

Similar to the visual system, auditory effort is normally associated with higher order cognitive functions such as attention, speech, and language comprehension (Peelle 2018). In contrast to the visual system, the auditory system is not a directional system in humans; we do not move our ears to hear a sound from a particular location in the same way as we move our eyes to see a target. Lack of a specialized “otomotor” system means a phenomenon similar to saccadic suppression does not explain effort perception in the auditory system. However, a principal idea of SAF is that suppression of self-generated sensory information is the basis of effort perception. Previous work shows that movement-induced sound modulation is robust (Reznik and Mukamel 2019). Self-generated sound refers to speech, or sounds produced by other moving body parts like pressing a button that emits a sound. Physiologically, self-generated auditory evoked potentials are attenuated and is mediated by the motor cortex and supplementary motor area (Reznik and others 2015b). Perceptually, the loudness can either be enhanced or attenuated with low sounds being amplified and high sounds being attenuated (Reznik and others 2015a).

Could poor movement-induced modulation of self-generated sounds explain fatigue? Although a possibility, it does not account for extreme fatigue when simply being (not interacting) in a noisy environment. This experience is suggestive of sensory overload unrelated to self-generated auditory input. Animal studies show that the motor system acts as a filter for auditory input irrespective of causality between motor action and auditory input. In mice, sounds that coincide with animal movement (and not triggered by the movement) is related to reduced sound-evoked local field potentials when compared to sounds presented at rest (Rummell and others 2016; Zhou and others 2014). The presence of anatomical connectivity between secondary motor areas and auditory association areas in mice (Nelson and others 2013; Schneider and others 2014) and nonhuman primates (Petrides and Pandya 2002) allows for the possibility of motor related suppression of auditory input. Therefore, SAF framework’s prediction of suppression of self-generated sounds as the basis of auditory effort perception must be altered to include suppression of all auditory input during motor cortex activation. Such poor auditory suppression may also directly stress the motor system by having to increase corticospinal output. Audio and visual distractors produce covert startle like response with greater cortico-muscular coherence required to maintain steady corticospinal output (Piitulainen and others 2015). Therefore, the lack of movement-induced auditory suppression might also make movement feel more effortful. In disease, the p3a component of auditory evoked potentials, a marker of higher order attentional orientation, is depressed and has longer latency in Parkinson’s fatigue (Pauletti and others 2019), while in MS fatigue the latency is shortened (Sandroni and others 1992). Both studies explicitly focused on later components (P3) of ERP; however, on closer examination of the raw data, earlier components (C1, P1, N1) normally associated with perception and sensory processing also appear altered. Direct evidence is needed to corroborate the predictions of SAF framework for auditory effort perception.

## A Comparison of the Models of Fatigue

**Figure 2.**
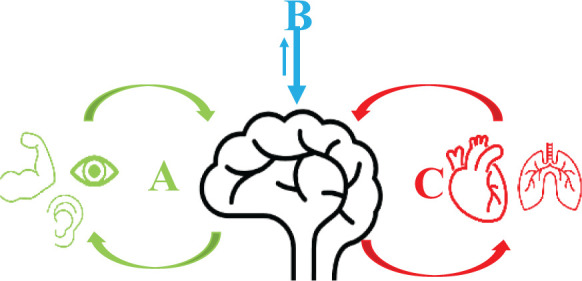
A schematic representation of the three models of fatigue: The sensory attenuation model of fatigue (A) is primarily based on poor exteroceptive sensory suppression as the basis of fatigue. The central sensitization model (B), on the other hand, relies on the overactivated inhibitory pathways in the brain as the basis of fatigue and the metacognitive model of fatigue (C) proposes that fatigue is a result of maladaptive interoceptive processing.

In this section, we identify commonalities and divergences of the SAF framework, the metacognitive theory of dyshomeostasis and central sensitization model (Fig. 2).

### Chronicity

While acute fatigue is a helpful indicator of an organism’s physical, cognitive, and emotional state, chronic fatigue is detrimental and it is important that any theory of chronic fatigue clearly identifies and discusses the origins of chronicity. SAF proposes that fatigue is maintained by continued altered sensorimotor interactions that underpins effort perception. The metacognitive theory explains chronicity as being triggered by dyshomeostasis, with repeated unfulfilled predictions resulting in reduced self-efficacy that continues after restoration of homeostasis. The central sensitization theory alludes to dysfunctional brain inhibitory mechanisms that continues long after the excitatory systems return to their normal state. While SAF and sensitization models both refer to dysfunction of relatively lower order brain function such as effort perception and alertness to explain chronicity, the metacognitive theory relies on dysfunction of higher order metacognitive dysfunction.

### External versus Internal Environment

A critical point of difference between the three theories is their differential focus on processing of information originating from outside or inside the body. The metacognitive and sensitization models focus on the internal environment and propose that fatigue is an experience of the internal state of the body. In contrast, SAF almost exclusively focusses on processing of information from the external environment. In acute fatigue, both internal and external information processing is altered. Loss of appetite, lack of motivation, reduced alertness relate to processing of internal stimuli while increased sensitivity to light and sound relate to external stimuli, with greater effort perception being associated with both internal and external stimuli. The metacognitive and sensitization models have dyshomeostasis as their fundamental premise of fatigue, focusing on metacognitive dysfunction and imbalance between the excitatory and inhibitory brain networks. Although neither theory speaks of high effort perception, a consequence of both dysfunctions is altered effort perception, a significant feature of fatigue. Altered effort perception, although influenced by the internal state of the body, SAF is mainly dependent on processing of external stimuli. For the special case of muscular effort perception where cardiovascular and respiratory modulators are the classical interoceptive elements, SAF predicts the processing of muscular afferent sensory information (the nonclassical interoceptive elements) has a greater role to play in altered muscular effort perception.

### Conscious Awareness

Fatigue is an experience; therefore, an inference and any process that contributes to fatigue must either be responsible for, or be closely linked to processes that underlie conscious awareness. In this view, the sensitization theory is unclear as to how altered inhibitory control results in fatigue, whereas the metacognitive and SAF models both explain how an experience of fatigue comes about, with SAF suggesting altered perception (metacognitive processing) of exteroceptive sensory information and the metacognitive theory alluding to altered perception of interoceptive information as the basis of fatigue.

## Does Evidence from Diseases Support the Models of Fatigue?

Is there direct experimental evidence in support of the models of fatigue? No. Nevertheless, results of fatigue related investigations in disease states can be understood from the viewpoint of the proposed models. Here I consider evidence from three major neurological diseases.

### Poststroke Fatigue

The vascular origins of stroke largely determines the pattern of deficits seen in stroke, with no particular vascular territory favoring the development of fatigue (Cumming and others 2016; Cumming and others 2018; Mead and others 2011). This favors a distributed network of brain regions that spans across different vascular territories that explains fatigue. The SAF framework posits that such a distributed network must be one that underpins perception of effort and work from my lab supports this notion. We showed that higher perceived effort explained greater trait fatigue (De Doncker and others 2020b) but not state fatigue, that is, the fatigue state at the time of task performance, indicating that such high perceived effort was not a consequence of fatigue but instead a driver of fatigue. Furthermore, a reduction in fatigue was accompanied by a reduction in perception of effort (De Doncker and others 2020c). The two investigations taken together supports the notion of altered effort perception drives pathological fatigue. Modafinil, a drug that interferes with reuptake of dopamine transporters, showed beneficial effects on fatigue in one study (Brioschi and others 2009) but not in another (Poulsen and others 2015). A more recent randomized controlled trial showed that modafinil is effective in reducing fatigue in some stroke survivors (Bivard and others 2017) with lower baseline functional connectivity between ipsilesional dorsolateral prefrontal cortex and contralesional thalamus and caudate predicting greater gains in fatigue reduction (Visser and others 2019). Dopamine significantly increases the willingness to expend effort for a given reward (Kurniawan and others 2010; Kurniawan and others 2011; Salamone and others 2016). However, there are no investigations addressing the effect of dopamine on effort perception. Could it be that dopamine-induced willingness to expend effort is due to a reduction in perception of effort (see later for discussion about dopamine and effort perception)? Or is it a result of greater reward affinity? If dopamine-induced change in effort-based decision making is mediated by altered effort perception, dopamine-induced reduction in fatigue is further evidence in support of the SAF framework.

Dopamine may act by influencing interoceptive networks with resulting change in allostatic self-efficacy as proposed by the metacognitive framework in relation to how dopamine may alleviate MS fatigue (Manjaly and others 2019). Dopaminergic system is an excitatory system and dopamine is unlikely to act by turning off overactivated inhibitory systems of the brain, thereby rejecting the inhibitory hypothesis of fatigue. However, neurophysiological investigations may provide some support for the inhibitory hypothesis of fatigue. Noninvasive brain stimulation studies show that higher the fatigue, lower is the motor cortical excitability at rest (Kuppuswamy and others 2015a), possibly reflecting greater underlying cortical inhibition. Not only is motor cortex excitability diminished at rest, but there is lesser pre-movement inhibition in fatigue (De Doncker and others 2020a). The state of excitability of the motor cortex prior to a movement is thought to be influenced by the level of uncertainty associated with the upcoming movement (Bestmann and Duque 2016; Bestmann and others 2008), with lesser inhibition associated with lesser uncertainty. In fatigue, the presence of lesser pre-movement inhibition may indicate a mismatch between true and predicted uncertainty associated with the upcoming task, leading to greater uncertainty and high perceived effort as proposed by SAF framework. Overall, based on the current state of evidence in poststroke fatigue the SAF framework has the most support, although it must be pointed out that, to date, there have been no prospectively designed investigations to address metacognitive experience of dyshomeostasis or overactive inhibition in poststroke fatigue.

### Multiple Sclerosis

This demyelinating disease has a very high prevalence of fatigue that is unrelated to disease burden of white matter lesion load and progression of disease (Ghajarzadeh and others 2013). Unlike stroke, the active disease process occurs over prolonged periods of time with inflammation being a large part of this process. The link between inflammation and fatigue is well-known and therefore the association between fatigue and hypothalamus-pituitary-adrenal axis function, central and peripheral inflammation have been addressed by several investigations (Akcali and others 2017; Gottschalk and others 2005; Heesen and others 2006). Surprisingly, very little inflammation associated processes explain fatigue in MS patients. On the other hand, emerging evidence from behavioral, neuroimaging and neurophysiological studies in MS fatigue suggests a neural network–level dysfunction that maintains long-term fatigue (Buyukturkoglu and others 2017; Chalah and others 2015; Engström and others 2013; Fiene and others 2018; Hidalgo de la Cruz and others 2017; Palotai and others 2019; Pravatà and others 2016; Shangyan and others 2018; Thickbroom and others 2006). Behaviorally, patients report lesser perceived effort (Heller and others 2016) with steeper fatigue-modulated increase in perceived effort in repeated tasks (Thickbroom and others 2006) supporting the SAF framework. Altered structural and functional connectivity at rest and during task performance also implicates several effort related brain regions. There is greater white matter microstructural damage in the cingulo-postcommissural-straito-thalamic, ventreromedial prefronto-postcommissural-striatal, and temporo-insular circuits in high fatigue, independent of total white matter lesion load (Palotai and others 2019). The regions include both subcortical sensorimotor integration areas and circuitry implicated in interoception lending support to both the SAF and dyshomeostasis hypotheses.

In resting state magnetic resonance imaging (MRI) studies, higher fatigue show greater frontostriatal connectivity, with increased connectivity between thalamus and sensorimotor cortex, and decreased connectivity with the insular cortex (Hidalgo de la Cruz and others 2017) implicating both sensorimotor and interoceptive circuitry. A resting state EEG (electroencephalogram) study showed a greater coherence in the beta band activity in the temporo-parietal network with greater fatigue (Buyukturkoglu and others 2017). Beta band activity is classically associated with motor execution and this motor-related resting state abnormality in fatigue suggests that despite no overt motor deficits, motor readiness may be altered in fatigue. While studies at rest showed that fatigue is primarily associated with networks involved in effort (scaling of physical effort) and in perception (both interoception and exteroception) such as the cortico-striato-thalamic circuit, cingulate, insular and parietal cortices; task-related studies showed that attentional networks are additionally modulated in fatigability protocols (Engström and others 2013; Spiteri and others 2019). Greater time on task was related to increased connectivity between superior frontal gyrus and temporal, frontal, and occipital lobes and subcortical structures such as the caudate in the high-fatigue group (Pravatà and others 2016). Therefore, while fatigue is mostly associated with altered perception, fatigability may primarily be a problem of greater demand on attentional resources.

There is an attenuation of pre-movement inhibition associated with greater MS fatigue (Morgante and others 2011), which can be similarly interpreted as with poststroke fatigue. Interventions targeting sensorimotor networks reduces fatigue (Cancelli and others 2018; Ferrucci and others 2014; Porcaro and others 2019; Tecchio and others 2014; Tecchio and others 2015); however, targeting the prefrontal and parietal attentional regions do not reduce fatigue (Ayache and Chalah 2018). Moreover, it has been shown that targeting the hand motor area (a standard target for motor cortex tDCS [transcranial direct current stimulation] interventions) is not effective in reducing fatigue (Ferrucci and others 2014; Tecchio and others 2015), while individualized anodal tDCS targeting whole body sensory cortex significantly reduces fatigue (Cancelli and others 2018; Tecchio and others 2014; Tecchio and others 2015). Such reduction appears to be via normalization of abnormal resting state intraregional connectivity within sensory cortex seen in high fatigue, specifically in the dominant hemisphere (Porcaro and others 2019). Previous findings of abnormal connectivity in dominant hemisphere is MS (Tecchio and others 2008), taken together with marked improvement in fatigue relating to connectivity changes in the dominant hemisphere suggests fatigue in MS is driven by network level dysfunction specifically in the sensory networks, lending strong support to the SAF model of fatigue. Modafinil also significantly reduced fatigue in MS patients (Shangyan and others 2018), supporting the dopamine dyshomeostasis theory, an influential neurochemical theory of fatigue (Dantzer and others 2014). Although dopamine’s role in fatigue has been explored from the perspective of reward-related motivation (Dantzer and others 2014; Dobryakova and others 2015) and interoceptive processing (Manjaly and others 2019), later in this article I discuss how dopamine’s role in sensory processing may be congruent with SAF predictions. Therefore, converging evidence from structural, functional, and interventional studies suggest that long-term maintenance of fatigue may be via poor sensory attenuation as hypothesized by the SAF framework. However, circuitry that are involved in exteroception are also implicated in interoception, and future work must aim to dissociate the roles of common circuitry in the various types of perception, so that sensible therapeutic targets can be developed.

### Parkinson’s Disease

Parkinson’s disease is a dopamine-responsive neurodegenerative disease that falls within the cluster of basal ganglia disorders characterized by distinctive motor deficits, with fatigue being a significant nonmotor symptom (Kluger 2017; Siciliano and others 2018) and severity of fatigue is unrelated to disease severity and motor deficits. A key pathology of Parkinson’s is the reduced availability of dopamine which responds to drugs that boost dopamine availability reducing motor symptoms. Then, why do dopaminergic drugs fail to alleviate fatigue (Elbers and others 2015)? Maybe fatigue is more strongly driven by network level dysfunction and not availability of a specific neurotransmitter. While direct evidence of altered effort perception is not available, studies investigating higher order cognitive dysfunction in fatigue may provide some support. In drug-naïve Parkinson’s patients, greater fatigue related to lower visuospatial perceptual ability (Kluger and others 2017) may possibly influence visual effort. Greater fatigue was also related to diminished auditory evoked potentials which may contribute to greater effort perception as discussed elsewhere in this article (Pauletti and others 2019).

At rest, higher fatigue related to reduced metabolic activity in the insula and superior temporal gyrus, greater activity in the posterior cingulate cortex, with altered connectivity between insula and somatosensory cortex, thalamus, motor, temporal, parietal, and prefrontal cortices (Cho and others 2017; Zhang and others 2017). Altered striatal activity and connectivity at rest, so heavily implicated in fatigue in both MS and stroke, is conspicuously absent from Parkinson’s resting state fatigue studies possibly due to not differentiating between levels of disease severity. A PET (positron emission tomography) study showed that nigrostriatal dopaminergic innervation predicted fatigue only in mild disease but not in moderate to severe disease (Chou and others 2016). Furthermore, gray matter volume in both caudate and putamen was correlated with fatigue levels (Kluger and others 2019). Despite structural investigations implicating basal ganglia in fatigue, functional studies do not. This could be a reflection of fatigue in Parkinson’s being triggered by the disease pathology, and yet maintenance of fatigue long term involves other functional circuits. Visual perceptual abnormalities and neural activity centered on sensorimotor neural structures may support the SAF framework; however, interoceptive abnormalities cannot be ruled out. There have been no systematic investigations linking autonomic or metacognitive dysfunction and fatigue.

### Dopamine and Fatigue

While network-level dysfunction in fatigue is the focus of this article, given the inextricable role of dopamine to many of the network-level dysfunction discussed here, I briefly describe how dopamine dyshomeostasis hypothesis of fatigue maybe in line with the network-level dysfunction of fatigue. Dopamine is a neuromodulatory monoamine, largely originating in the midbrain, with extensive cortical and subcortical distribution and a primary function of reward-related signaling in frontostriatal circuitry. In reward-based choice tasks, dopaminergic activity signals if the effort is worth the reward, thereby encoding the worth of reward. This, along with the effectiveness of dopamine in modulating fatigue in disease conditions, suggests dopamine may reduce fatigue by altering how an effort is perceived given a fixed reward. While the idea of dopamine-induced change in perceived effort fits the SAF framework, dopamine’s role in altering reward value (increased motivation) may not be in line with SAF predictions; however, a lesser studied role of dopamine in sensory perception (Jacob and Nienborg 2018; Perelmuter and others 2019; Valdés-Baizabal and others 2020; Yousif and others 2016) may explain altered perception. Animal studies show that dopamine directly impacts on perception of visual, auditory, and somatosensory stimuli, specifically, the effect of dopamine on signal-to-noise ratio in auditory discrimination tasks lends itself to explaining fatigue-related altered perception in the context of SAF. A third possible method of action could be by altering affect but not the sensation of fatigue. It has been seen that depletion of dopamine precursors can reduce the unpleasantness of a pain stimulus without changing the sensation of pain (Tiemann and others 2014). Similar mechanisms may play a role in fatigue, but this is yet to be confirmed.

## Where, and How Is the Brain Involved in Fatigue Generation?

**Figure 3.**
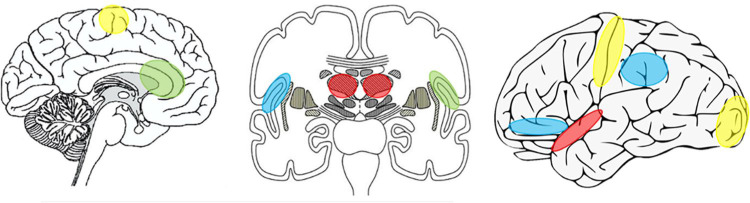
Activity in brain regions that covary with self-reported effort levels: Sensory processing and integration areas (thalamus, superior temporal gyrus) in red, ventral attentional regions (inferior parietal lobe, inferior frontal gyrus and anterior insula) in blue, interoceptive and awareness areas (anterior insula and anterior cingulate cortex) in green, and modality specific representations (sensorimotor cortex, SMA, and occipital cortex) in yellow.

In this section, I focus on the neural origins of fatigue as predicted by the SAF framework. Perception of effort has been largely studied in the motor system, in the context of a motor task or repetition of a motor task, where both peripheral and central factors contribute to perceived effort (Fig. 3). The influence of peripheral factors has been investigated using several methods including sensory afferent blocks, tendon vibration, and exercise-induced changes in afferent input, while maintaining the motor output. Such peripheral factors and their role in effort perception have been reviewed extensively elsewhere (Cos 2017; Lafargue and Franck 2009; Lafargue and others 2003; Lafargue and others 2006; Philbeck and Witt 2015; Proske and Allen 2019; Salamone and others 2016). Here, the focus is on brain regions whose activity underpin effort perception, the anatomical connectivity of these regions and the architectural features that allow for alteration of gain, a key requirement for a psychophysical experience, such as effort perception. Understanding functional activity in light of the structural substrate and its architecture will help us speculate on the key central generator(s) and modulators of effort perception.

### Neural Correlates of Effort Perception

Effort and its subjective value, role in decision making, interaction with reward, impact on motor performance, and representation in the brain are very well studied (Cos 2017; de Morree and others 2014; Hogan and others 2019; Klein-Flügge and others 2015; Kurniawan and others 2010; Kurniawan and others 2011; Kurniawan and others 2013; Westbrook and others 2019). However, very few studies have focused on the brain correlates of self-reported effort, that is, effort perception. The few that have, use a motor task to manipulate effort levels, with one study focusing on cognitive effort. The role of motor cortex in perception of effort has been delineated from its role in motor output in an EEG study where the early components of motor related cortical potentials (MRCP) track perceived effort but not the actual motor force output(Slobounov and others 2004). Motor corollaries thought to have a role in sensory predictions maybe reflected in the early components of MRCP. Motor cortex “lesion” studies show that disrupting motor cortex results in a performance change in a task that relies on perceived effort (Takarada and others 2014), although a direct measure of perceived effort was not used. Moving further upstream, disruption of SMA significantly decreases perceived effort both in self-reported measures and performance based measure (Zénon and others 2015). Involvement of sensory and higher order integration areas in fatigue was seen in a study where visual feedback was used to manipulate perceived effort while motor performance was maintained constant. They showed that while effort perception did not track changes in heart rate and oxygen consumption, which more closely reflected the motor output, greater effort perception was associated with increased activation of right thalamus and insula and a decrease with reduced anterior cingulate cortex and left insula activation (Williamson and others 2001). A study evaluating both task difficulty and perceived effort in a cognitive task showed that the left anterior insula, inferior frontal gyrus, thalamus, the right inferior parietal sulcus, bilateral occipital gyrus, and the left superior temporal sulcus were all more active during evaluation of effort perception than during task difficulty evaluation. Task difficulty evaluation additionally activated several regions of the basal ganglia (Otto and others 2014). Taken together, the brain regions whose activity co-varies with effort perception include the sensorimotor cortices, thalamus, anterior insula, superior temporal sulcus/gyrus, anterior cingulate cortex, inferior frontal gyrus, and inferior parietal lobe. These effort perception-associated brain regions significantly overlap with regions implicated in pathological fatigue, as discussed earlier. Additional areas related to fatigue but not effort perception are posterior cingulate cortex, caudate, and putamen, whose role in fatigue is yet to be explained.

Brain areas whose activity tracks self-reported effort levels can be classified into four categories. Sensory processing and integration areas (thalamus, superior temporal gyrus), ventral attentional regions (inferior parietal lobe, inferior frontal gyrus, and anterior insula), interoceptive and awareness areas (anterior insula and anterior cingulate cortex), and modality specific representations (sensorimotor cortex, SMA, and occipital cortex). While neural networks involved in effort perception is task-dependent, there may yet be task-independent contributors to effort perception. There is no direct evidence for this claim; however, neuronal architecture and connectivity might suggest this. The thalamus is a major sensory processing hub of the brain where all sensory input, except olfaction, converge on entry into the central nervous system. The thalamus has extensive cortical, and cerebellar projections, and while once thought of as simply a sensory relay station, we now know that complex sensory gating and modulation of sensory gain occurs within the thalamus (Halassa and Sherman 2019; Sherman 2017). The synaptic architecture of thalamic neurons with multiple inputs synapsing on to the dendrites of the output neurons, some with modulatory GABAergic projections, makes the thalamus a strong candidate region for gain modulation of incoming sensory information. Modulatory GABAergic inputs include projections from the basal ganglia, a structure heavily implicated in encoding motor vigor (Bolam and others 2000). With its property of gain modulation the thalamus has a significant role in effort perception, irrespective of the nature of task involved. The basal ganglia with its known role in motor effort representation may be involved in effort perception in motor tasks; however, its anatomical connectivity to other higher order sensory cortices, along with its close connections to the thalamus, may suggest a role outside of motor effort perception.

The insula, with its role in awareness of internal state of the body is likely to play a role in effort perception by signaling the homeostatic state of body, especially in conditions such as exercise induced state of exhaustion. However, beyond homeostatic signaling, the extensive connectivity of posterior insula with posterior temporal, parietal, and sensorimotor areas (Craig 2009; Uddin and others 2017) and its role in exteroception suggests the insula may also inform effort perception by exteroceptive sensory processing. Another medial cortical structure with extensive anatomical connectivity is the anterior cingulate cortex (Heilbronner and Hayden 2016) that subserves higher order cognitive functions may inform effort perception by signaling high complexity and sustained attentional needs. Other cortical regions implicated include the superior temporal gyrus and sulcus, inferior frontal gyrus, and inferior parietal lobe. The STG is a multisensory integration area with roles in auditory processing alongside language comprehension and complex behavioral traits (Beauchamp 2005; Friederici 2011) suggesting contribution to effort perception specifically when task requirements are complex. The inferior frontal gyrus with its long range connections to the frontal, temporal, and parietal cortices (Briggs and others 2019), along with the inferior parietal lobe and neighboring superior temporal regions, form the ventral attentional network commonly thought to be involved in bottom-up attention. This indicates that stimulus driven processes are significant for effort perception irrespective of higher order task complexity.

The role of higher order sensorimotor regions in effort perception can be inferred from the perspective of SAF framework, as regions that generate sensory predictions that are essential for modulation of ascending prediction errors, the process whose psychophysical output is effort perception. To summarize, the regions implicated during effort perception includes those that are involved in stimulus driven sensory processing, those that set sensory gain and other regions involved in higher order attention, executive function, and homeostatic inference. The regions involved in bottom-up sensory processing and higher order sensory areas that set top-down gain can been seen as the primary network generating effort perception, with attentional, executive, and homeostatic regions being secondary, task-specific top-down modulators of effort perception. As for pathological fatigue, the SAF framework predicts that fatigue is a result of greater effort perception driven by poor sensory attenuation, attributed to either incorrect sensory predictions or abnormal ascending predictor errors. Evidence thus far from health and disease suggest there is a significant overlap between areas implicated in effort perception and fatigue with interventional paradigms supporting the SAF framework; however, these studies do no exclude fatigue from being an attentional or higher order executive disorder or indeed a disorder of homeostasis.

## Sensory Attenuation, Sense of Agency, Effort, and Fatigue

**Figure 4.**
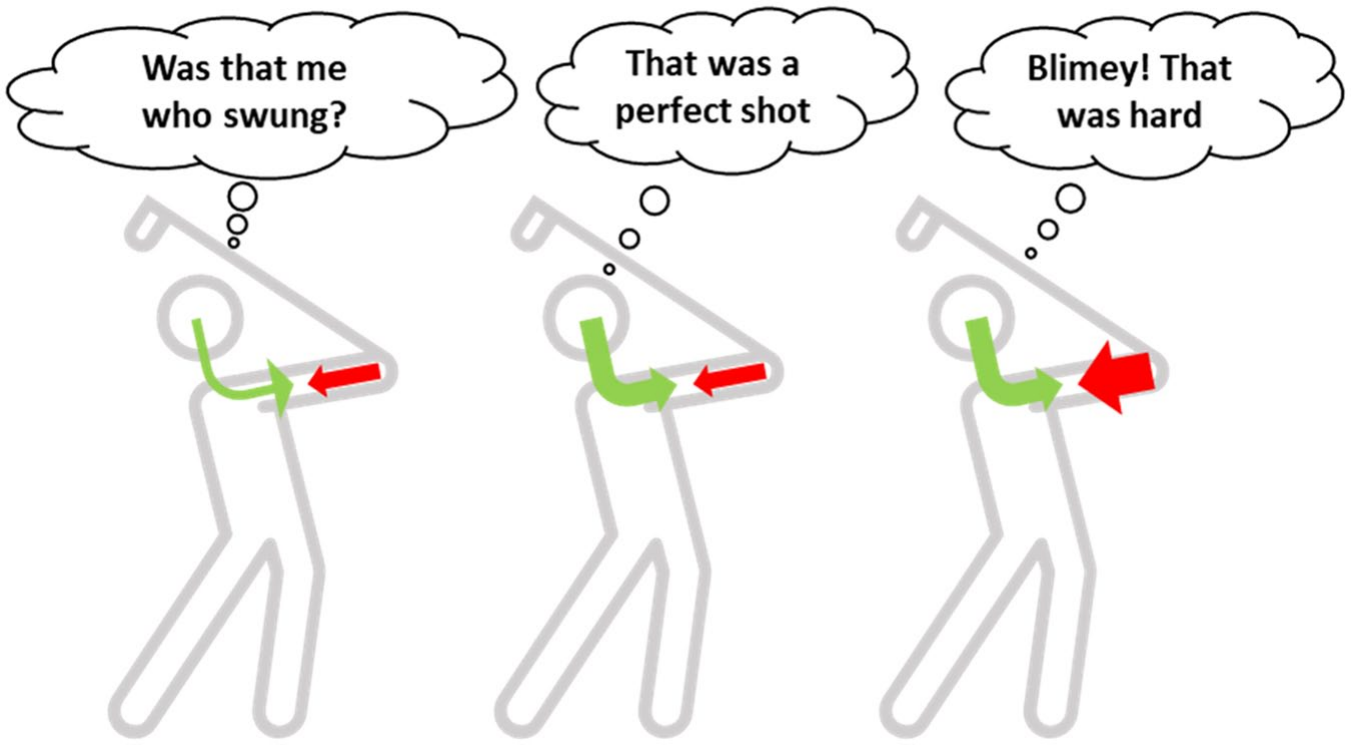
An illustration of the link between sensory attenuation, agency, and fatigue: When the sensory input is sufficiently suppressed, the resultant feeling of full control over the consequences leads to a strong sense of agency (middle golfer). However, if the predictions do not match, the resulting experience is varied. Here I hypothesize that poor predictions may result in altered sense of agency (left golfer), while greater gain of prediction errors may cause the feeling of effort and fatigue (right golfer).

A key question that arises in relation to the SAF framework of pathological fatigue is—How does poor sensory attenuation, a fundamental deficit seen in disorders of agency compatible with the idea of such deficit also underlying fatigue (Fig. 4)? Do those with agency disorders also exhibit fatigue? Is there any evidence for alterations in sense of agency in those with fatigue? The classic case of altered sense of agency is seen in schizophrenia where patients often attribute sensory consequences to an external agent (Brown and others 2013). To experience a sense of agency, one must have a feeling of exerting control over an action, and hence perceiving exertion or effort perception plays an important role, with greater effort being associated with higher sense of agency (Chambon and others 2014). In schizophrenia it is thought that changes in effort perception might underlie distortion of agency, explained by a partial or total absence of sensory predictions (Lafargue and Franck 2009). Another group of disorders where there is altered sense of agency that has been attributed to poor sensory attenuation is functional neurological disorders (Edwards and others 2013). Both schizophrenia and FND exhibit complex neurocognitive and affective symptoms including fatigue; however, fatigue is not the defining feature of the disorders. In those diseases where fatigue is a significant symptom, there is little evidence for alterations in agency; however, there have been reports of loss of control and the body being described as a heavy object, possibly delineating the body from the self (Whitehead and others 2016). Such experience has been seen in a wide variety of diseases including cancer, neurological illnesses, cardiovascular diseases, and neuromuscular disorders, suggesting these experiences are not disease specific, but a feature of long-standing fatigue. Specific deficits such as body heaviness that may interfere with experience of the physical self may contribute to the sense of agency. Body heaviness has previously been thought of as a result of poor sensory suppression of muscle afferent information arising from resting muscle tone (Kuppuswamy and others 2016). Whether this is a result of poor sensory predictions (descending) or prediction errors (ascending) is still an open question. In fact, the difference in reported symptomology of “external control” versus “body heaviness” may both be a result of poor sensory attenuation, but driven by different deficits, one by poor predictions and other by abnormal prediction errors. In this view, fatigue could be placed within the spectrum of agency disorders. Another typical behavior associated with fatigue is the greatly reduced amount of voluntary activity; however, it is unclear if this is a result of disturbance in volition per se or a fatigue induced reduction in motivation leading to reduced voluntary activity. Volition is inextricably linked to sense of agency, with intact sense of agency being a pre-requisite for volition (Chambon and others 2014; Kranick and Hallett 2013). If future studies in fatigue confirm a disturbance in volition, it further strengthens the idea of fatigue as a disorder of agency.

## Conclusions

The need for research into pathological fatigue is at a tipping point, with increasing recognition of fatigue as a primary and significant problem both by patients and health care professionals, and the notion of fatigue being a significant risk factor for several mental health problems. However, research into pathological fatigue is still rudimentary with not enough evidence in support of an overarching fatigue mechanism(s) that can then form the basis of effective interventions. The last decade has seen the emergence of few mechanistic theories that attempt to explain the cause of fatigue that is irreversible and seemingly without a cause. These include the dyshomeostasis theories of interoceptive processing and overactivated inhibitory systems, and the sensory attenuation model of fatigue. While there is fragmented evidence across diseases that support the proposed theories, some more than others, robust large-scale studies spanning different conditions is absolutely vital. The evidence for brain-based mechanisms is greater in neurological conditions; however, it is important that brain-based mechanisms are explored in more depth in non-neurological conditions, to establish disease-independent mechanisms of fatigue. While homeostatic causes of fatigue have been implicitly agreed upon in years of fatigue research and fatigue management, the exteroceptive theory of poor sensory attenuation is a new perspective on fatigue, with some prospective observational and interventional studies providing solid evidence in neurological conditions. Crucially, this framework allows us to explain more of the defining features of chronic fatigue such as high perceived effort, greater exteroceptive sensitivity, loss of control, and altered bodily sense such as heaviness. Moreover, this framework is also useful to explain fatigue when there is little homeostatic cause for fatigue, as is the case with most long-term fatigue. With new theories laying the foundation for hereto unexplained aspects of pathological fatigue, new opportunities arise to understand fatigue across various diseases. Future fatigue research must focus on exploring exteroceptive sensory processing that underlie effort perception, as the primary cause of long-term maintenance of fatigue.

## References

[bibr1-1073858420985447] Akcali A Zengin F Aksoy SN Zengin O. 2017. Fatigue in multiple sclerosis: is it related to cytokines and hypothalamic-pituitary-adrenal axis? Mult Scler Relat Disord 15:37–41. 28641771 10.1016/j.msard.2017.03.004

[bibr2-1073858420985447] Ament W Verkerke GJ . 2009. Exercise and fatigue. Sports Med 39:389–422. 19402743 10.2165/00007256-200939050-00005

[bibr3-1073858420985447] Ayache SS Chalah MA . 2018. Transcranial direct current stimulation: a glimmer of hope for multiple sclerosis fatigue? J Clin Neurosci 55:10–2. 10.1016/j.jocn.2018.06.002 29914773

[bibr4-1073858420985447] Barbour VL Mead GE . 2012. Fatigue after stroke: the patient’s perspective. Stroke Res Treat 2012:863031. 21860808 10.1155/2012/863031 PMC3154793

[bibr5-1073858420985447] Beauchamp MS . 2005. See me, hear me, touch me: multisensory integration in lateral occipital-temporal cortex. Curr Opin Neurobiol 15:145–53. 10.1016/j.conb.2005.03.011 15831395

[bibr6-1073858420985447] Bestmann S Duque J. 2016. Transcranial magnetic stimulation: decomposing the processes underlying action preparation. Neuroscientist 22:392–405. 26163320 10.1177/1073858415592594

[bibr7-1073858420985447] Bestmann S Harrison LM Blankenburg F Mars RB Haggard P Friston KJ , and others. 2008. Influence of uncertainty and surprise on human corticospinal excitability during preparation for action. Curr Biol 18:775–80. 10.1016/j.cub.2008.04.051 PMC2387198 18485711

[bibr8-1073858420985447] Bivard A Lillicrap T Krishnamurthy V Holliday E Attia J Pagram H , and others. 2017. MIDAS (Modafinil in Debilitating Fatigue After Stroke): a randomized, double-blind, placebo-controlled, cross-over trial. Stroke 48:1293–8. 10.1161/STROKEAHA.116.016293 PMC5404401 28404841

[bibr9-1073858420985447] Bolam JP Hanley JJ Booth PAC Bevan MD . 2000. Synaptic organisation of the basal ganglia. J Anat 196:527–42. 10.1046/j.1469-7580.2000.19640527.x PMC1468095 10923985

[bibr10-1073858420985447] Bridgeman B . 2005. Influence of visually induced expectation on perceived motor effort: a visual-proprioceptive interaction at the Santa Cruz Mystery Spot. Psychonom Bull Rev 12:549–52. 10.3758/bf03193803 16235644

[bibr11-1073858420985447] Briggs RG Chakraborty AR Anderson CD Abraham CJ Palejwala AH Conner AK , and others. 2019. Anatomy and white matter connections of the inferior frontal gyrus. Clin Anat 32:546–56. 10.1002/ca.23349 30719769

[bibr12-1073858420985447] Brioschi A Gramigna S Werth E Staub F Ruffieux C Bassetti C , and others. 2009. Effect of modafinil on subjective fatigue in multiple sclerosis and stroke patients. Eur Neurol 62:243–9. 10.1159/000232927 19672078

[bibr13-1073858420985447] Brooks J Allen TJ Proske U. 2013. The senses of force and heaviness at the human elbow joint. Exp Brain Res 226: 617–29. 10.1007/s00221-013-3476-6 23525562

[bibr14-1073858420985447] Brooks JX Cullen KE . 2019. Predictive sensing: the role of motor signals in sensory processing. Biol Psychiatry Cogn Neurosci Neuroimaging 4:842–50. 10.1016/j.bpsc.2019.06.003 PMC6733654 31401034

[bibr15-1073858420985447] Brown H Adams RA Parees I Edwards M Friston K. 2013. Active inference, sensory attenuation and illusions. Cogn Process 14:411–27. 10.1007/s10339-013-0571-3 PMC3824582 23744445

[bibr16-1073858420985447] Buyukturkoglu K Porcaro C Cottone C Cancelli A Inglese M Tecchio F. 2017. Simple index of functional connectivity at rest in multiple sclerosis fatigue. Clin Neurophysiol 128:807–13. 10.1016/j.clinph.2017.02.010 28340429

[bibr17-1073858420985447] Cancelli A Cottone C Giordani A Migliore S Lupoi D Porcaro C , and others. 2018. Personalized, bilateral whole-body somatosensory cortex stimulation to relieve fatigue in multiple sclerosis. Mult Scler 24:1366–74. 10.1177/1352458517720528 28756744

[bibr18-1073858420985447] Chalah MA Ayache SS . 2018. Is there a link between inflammation and fatigue in multiple sclerosis? J Inflamm Res 11:253–64. 10.2147/JIR.S167199 PMC5995280 29922081

[bibr19-1073858420985447] Chalah MA Riachi N Ahdab R Créange A Lefaucheur JP Ayache SS . 2015. Fatigue in multiple sclerosis: neural correlates and the role of non-invasive brain stimulation. Front Cell Neurosci 9:460. 26648845 10.3389/fncel.2015.00460 PMC4663273

[bibr20-1073858420985447] Chambon V Sidarus N Haggard P. 2014. From action intentions to action effects: how does the sense of agency come about? Front Hum Neurosci 8:320. 24860486 10.3389/fnhum.2014.00320 PMC4030148

[bibr21-1073858420985447] Chaudhuri A Behan PO . 2004. Fatigue in neurological disorders. Lancet 363:978–88. 10.1016/S0140-6736(04)15794-2 15043967

[bibr22-1073858420985447] Cho SS Aminian K Li C Lang AE Houle S Strafella AP . 2017. Fatigue in Parkinson’s disease: the contribution of cerebral metabolic changes. Hum Brain Mapp 38:283–92. 10.1002/hbm.23360 PMC6867035 27571419

[bibr23-1073858420985447] Chou KL Kotagal V Bohnen NI . 2016. Neuroimaging and clinical predictors of fatigue in Parkinson disease. Parkinsonism Relat Disord 23:45–9. 10.1016/j.parkreldis.2015.11.029 PMC4724499 26683744

[bibr24-1073858420985447] Christensen D Johnsen SP Watt T Harder I Kirkevold M Andersen G. 2008. Dimensions of post-stroke fatigue: a two-year follow-up study. Cerebrovasc Dis 26:134–41. 10.1159/000139660 18560216

[bibr25-1073858420985447] Cos I . 2017. Perceived effort for motor control and decision-making. PLoS Biol 15:e2002885. 29166398 10.1371/journal.pbio.1002617 PMC5699818

[bibr26-1073858420985447] Craig ADB . 2009. How do you feel—now? The anterior insula and human awareness. Nat Rev Neurosci 10:59–70. 19096369 10.1038/nrn2555

[bibr27-1073858420985447] Cumming TB Packer M Kramer SF English C. 2016. The prevalence of fatigue after stroke: a systematic review and meta-analysis. Int J Stroke 11:968–77. 10.1177/1747493016669861 27703065

[bibr28-1073858420985447] Cumming TB Yeo AB Marquez J Churilov L Annoni JM Badaru U , and others. 2018. Investigating post-stroke fatigue: an individual participant data meta-analysis. J Psychosom Res 113:107–12. 10.1016/j.jpsychores.2018.08.006 30190042

[bibr29-1073858420985447] Dantzer R Heijnen CJ Kavelaars A Laye S Capuron L. 2014. The neuroimmune basis of fatigue. Trends Neurosci 37:39–46. 24239063 10.1016/j.tins.2013.10.003 PMC3889707

[bibr30-1073858420985447] De Doncker W Brown K Kuppuswamy A . 2020a. Influence of fatigue on reaction time and corticospinal excitability during movement preparation. Clin Neurophysiol 132: 191–9. 10.1016/j.clinph.2020.11.012 PMC7810236 33302061

[bibr31-1073858420985447] De Doncker W Charles L Ondobaka S Kuppuswamy A . 2020b. Exploring the relationship between effort perception and post-stroke fatigue. Neurology 95:e3321–e3330. 10.1212/WNL.0000000000010985 PMC7836654 33067406

[bibr32-1073858420985447] De Doncker W Dantzer R Ormstad H Kuppuswamy A . 2018. Mechanisms of poststroke fatigue. J Neurol Neurosurg Psychiatry 89:287–93. 10.1136/jnnp-2017-316007 28939684

[bibr33-1073858420985447] De Doncker W Ondobaka S Kuppuswamy A . 2020c. Effect of tDCS on effort perception and post-stroke fatigue. J Neurol (under review). DOI: 10.1101/2020.11.18.20227272

[bibr34-1073858420985447] de Morree HM Klein C Marcora SM . 2012. Perception of effort reflects central motor command during movement execution. Psychophysiology 49:1242–53. 10.1111/j.1469-8986.2012.01399.x 22725828

[bibr35-1073858420985447] de Morree HM Klein C Marcora SM . 2014. Cortical substrates of the effects of caffeine and time-on-task on perception of effort. J Appl Physiol 117:1514–23. 10.1152/japplphysiol.00898.2013 25342703

[bibr36-1073858420985447] Dell’Acqua ML Landi D Zito G Zappasodi F Lupoi D Rossini PM , and others. 2010. Thalamocortical sensorimotor circuit in multiple sclerosis: an integrated structural and electrophysiological assessment. Hum Brain Mapp 31:1588–600. 10.1002/hbm.20961 PMC6871076 20162580

[bibr37-1073858420985447] Dobryakova E Genova HM DeLuca J Wylie GR . 2015. The dopamine imbalance hypothesis of fatigue in multiple sclerosis and other neurological disorders. Front Neurol 6:52. 25814977 10.3389/fneur.2015.00052 PMC4357260

[bibr38-1073858420985447] Dong W Yan B Johnson BP Millist L Davis S Fielding J , and others. 2013. Ischaemic stroke: the ocular motor system as a sensitive marker for motor and cognitive recovery. J Neurol Neurosurg Psychiatry 84:337–41. 10.1136/jnnp-2012-303926 PMC3582066 23223333

[bibr39-1073858420985447] Edwards MJ Fotopoulou A Pareés I. 2013. Neurobiology of functional (psychogenic) movement disorders. Curr Opin Neurol 26:442–7. 10.1097/WCO.0b013e3283633953 PMC4196785 23823467

[bibr40-1073858420985447] Elbers RG Verhoef J van Wegen EE Berendse HW Kwakkel G. 2015. Interventions for fatigue in Parkinson’s disease. Cochrane Database Syst Rev (10):CD010925. 26447539 10.1002/14651858.CD010925.pub2 PMC9240814

[bibr41-1073858420985447] Engbert R . 2006. Microsaccades: a microcosm for research on oculomotor control, attention, and visual perception. Prog Brain Res 154:177–92. 10.1016/S0079-6123(06)54009-9 17010710

[bibr42-1073858420985447] Engström M Flensner G Landtblom AM Ek AC Karlsson T. 2013. Thalamo-striato-cortical determinants to fatigue in multiple sclerosis. Brain Behav 3:715–28. 10.1002/brb3.181 PMC3868176 24363974

[bibr43-1073858420985447] Felger JC Miller AH . 2012. Cytokine effects on the basal ganglia and dopamine function: the subcortical source of inflammatory malaise. Front Neuroendocrinol 33:315–27. 10.1016/j.yfrne.2012.09.003 PMC3484236 23000204

[bibr44-1073858420985447] Ferreira M Pereira PA Parreira M Sousa I Figueiredo J Cerqueira JJ , and others. 2017. Using endogenous saccades to characterize fatigue in multiple sclerosis. Mult Scler Relat Disord 14:16–22. 28619425 10.1016/j.msard.2017.01.014

[bibr45-1073858420985447] Ferrucci R Vergari M Cogiamanian F Bocci T Ciocca M Tomasini E , and others. 2014. Transcranial direct current stimulation (tDCS) for fatigue in multiple sclerosis. Neurorehabilitation 34:121–7. 10.3233/NRE-131019 24284464

[bibr46-1073858420985447] Fiene M Rufener KS Kuehne M Matzke M Heinze HJ Zaehle T. 2018. Electrophysiological and behavioral effects of frontal transcranial direct current stimulation on cognitive fatigue in multiple sclerosis. J Neurol 265:607–17. 10.1007/s00415-018-8754-6 29356975

[bibr47-1073858420985447] Finke C Pech LM Sömmer C Schlichting J Stricker S Endres M , and others. 2012. Dynamics of saccade parameters in multiple sclerosis patients with fatigue. J Neurol 259:2656–63. 10.1007/s00415-012-6565-8 22711158

[bibr48-1073858420985447] Flinn NA Stube JE . 2010. Post-stroke fatigue: qualitative study of three focus groups. Occup Ther Int 17:81–91. 19787634 10.1002/oti.286

[bibr49-1073858420985447] Friederici AD . 2011. The brain basis of language processing: from structure to function. Physiol Rev 91:1357–92. 10.1152/physrev.00006.2011 22013214

[bibr50-1073858420985447] Gandevia SC . 1982. The perception of motor commands or effort during muscular paralysis. Brain 105:151–9. 10.1093/brain/105.1.151 6461386

[bibr51-1073858420985447] Ghajarzadeh M Jalilian R Eskandari G Sahraian MA Azimi A Mohammadifar M. 2013. Fatigue in multiple sclerosis: relationship with disease duration, physical disability, disease pattern, age and sex. Acta Neurol Belg 113:411–4. 10.1007/s13760-013-0198-2 23616230

[bibr52-1073858420985447] Gottschalk M Kümpfel T Flachenecker P Uhr M Trenkwalder C Holsboer F , and others. 2005. Fatigue and regulation of the hypothalamo-pituitary-adrenal axis in multiple sclerosis. Arch Neurol 62:277–80. 10.1001/archneur.62.2.277 15710856

[bibr53-1073858420985447] Hagelin CL Wengström Y Ahsberg E Fürst CJ . 2009. Fatigue dimensions in patients with advanced cancer in relation to time of survival and quality of life. Palliat Med 23:171–8. 10.1177/0269216308098794 18952749

[bibr54-1073858420985447] Halassa MM Sherman SM . 2019. Thalamocortical circuit motifs: a general framework. Neuron 103:762–70. 10.1016/j.neuron.2019.06.005 PMC6886702 31487527

[bibr55-1073858420985447] Haroon E Miller AH Sanacora G. 2017. Inflammation, glutamate, and glia: a trio of trouble in mood disorders. Neuropsychopharmacology 42:193–215. 27629368 10.1038/npp.2016.199 PMC5143501

[bibr56-1073858420985447] Heesen C Nawrath L Reich C Bauer N Schulz KH Gold SM . 2006. Fatigue in multiple sclerosis: an example of cytokine mediated sickness behaviour? J Neurol Neurosurg Psychiatry 77:34–9. 10.1136/jnnp.2005.065805 PMC2117393 16361589

[bibr57-1073858420985447] Heilbronner SR Hayden BY . 2016. Dorsal anterior cingulate cortex: a bottom-up view. Annu Rev Neurosci 39:149–70. 10.1146/annurev-neuro-070815-013952 PMC5512175 27090954

[bibr58-1073858420985447] Heller M Retzl I Kiselka A Greisberger A. 2016. Perception of muscular effort during dynamic elbow extension in multiple sclerosis. Arch Phys Med Rehabil 97:252–8. 10.1016/j.apmr.2015.10.082 26525526

[bibr59-1073858420985447] Helmchen C Pohlmann J Trillenberg P Lencer R Graf J Sprenger A. 2012. Role of anticipation and prediction in smooth pursuit eye movement control in Parkinson’s disease. Mov Disord 27:1012–8. 10.1002/mds.25042 22693071

[bibr60-1073858420985447] Hewlett S Dures E Almeida C. 2011. Measures of fatigue: Bristol Rheumatoid Arthritis Fatigue Multi-Dimensional Questionnaire (BRAF MDQ), Bristol Rheumatoid Arthritis Fatigue Numerical Rating Scales (BRAF NRS) for severity, effect, and coping, Chalder Fatigue Questionnaire (CFQ), Checklist Individual Strength (CIS20R and CIS8R), Fatigue Severity Scale (FSS), Functional Assessment Chronic Illness Therapy (Fatigue) (FACIT-F), Multi-Dimensional Assessment of Fatigue (MAF), Multi-Dimensional Fatigue Inventory (MFI), Pediatric Quality of Life (PedsQL) Multi-Dimensional Fatigue Scale, Profile of Fatigue (ProF), Short Form 36 Vitality Subscale (SF-36 VT), and Visual Analog Scales (VAS). Arthritis Care Res (Hoboken) 63(Suppl 11):S263–S286. 22588750 10.1002/acr.20579

[bibr61-1073858420985447] Hidalgo de la Cruz M d’Ambrosio A Valsasina P Pagani E Colombo B Rodegher M , and others. 2017. Abnormal functional connectivity of thalamic sub-regions contributes to fatigue in multiple sclerosis. Mult Scler 24(9):1183–95. 10.1177/1352458517717807 28657428

[bibr62-1073858420985447] Hogan PS Galaro JK Chib VS . 2019. Roles of ventromedial prefrontal cortex and anterior cingulate in subjective valuation of prospective effort. Cereb Cortex 29:4277–90. 10.1093/cercor/bhy310 PMC6735256 30541111

[bibr63-1073858420985447] Hughes AJ Bhattarai JJ Paul S Beier M. 2019. Depressive symptoms and fatigue as predictors of objective-subjective discrepancies in cognitive function in multiple sclerosis. Mult Scler Relat Disord 30:192–7. 10.1016/j.msard.2019.01.055 PMC7282884 30797133

[bibr64-1073858420985447] Jacob SN Nienborg H. 2018. Monoaminergic neuromodulation of sensory processing. Front Neural Circuits 12:51. 30042662 10.3389/fncir.2018.00051 PMC6048220

[bibr65-1073858420985447] Joyner MJ . 2016. Fatigue: where did we come from and how did we get here? Med Sci Sports Exer 48:2224–7. 10.1249/MSS.0000000000000938 27031741

[bibr66-1073858420985447] Klein-Flügge MC Kennerley SW Saraiva AC Penny WD Bestmann S. 2015. Behavioral modeling of human choices reveals dissociable effects of physical effort and temporal delay on reward devaluation. PLoS Comput Biol 11:e1004116. 25816114 10.1371/journal.pcbi.1004116 PMC4376637

[bibr67-1073858420985447] Kluger BM . 2017. Fatigue in Parkinson’s disease. Int Rev Neurobiol 133:743–68. 10.1016/bs.irn.2017.05.007 28802940

[bibr68-1073858420985447] Kluger BM Pedersen KF Tysnes OB Ongre SO Øygarden B Herlofson K. 2017. Is fatigue associated with cognitive dysfunction in early Parkinson’s disease? Parkinsonism Relat Disord 37:87–91. 28202373 10.1016/j.parkreldis.2017.02.005

[bibr69-1073858420985447] Kluger BM Zhao Q Tanner JJ Schwab NA Levy SA Burke SE , and others. 2019. Structural brain correlates of fatigue in older adults with and without Parkinson’s disease. Neuroimage Clin 22:101730. 30818269 10.1016/j.nicl.2019.101730 PMC6396012

[bibr70-1073858420985447] Kranick SM Hallett M. 2013. Neurology of volition. Exp Brain Res 229:313–27. 10.1007/s00221-013-3399-2 PMC4744643 23329204

[bibr71-1073858420985447] Kuppuswamy A . 2017. The fatigue conundrum. Brain 140:2240–5. 10.1093/brain/awx153 PMC5806506 28899013

[bibr72-1073858420985447] Kuppuswamy A Clark E Rothwell J Ward NS . 2016. Limb heaviness: a perceptual phenomenon associated with poststroke fatigue? Neurorehabil Neural Repair 30:360–2. 10.1177/1545968315597071 26187642

[bibr73-1073858420985447] Kuppuswamy A Clark EV Turner IF Rothwell JC Ward NS . 2015a. Post-stroke fatigue: a deficit in corticomotor excitability? Brain 138:136–48. 10.1093/brain/awu306 PMC4441078 25367024

[bibr74-1073858420985447] Kuppuswamy A Rothwell J Ward N. 2015b. A model of poststroke fatigue based on sensorimotor deficits. Curr Opin Neurol 28:582–6. 10.1097/WCO.0000000000000260 26397231

[bibr75-1073858420985447] Kurniawan IT Guitart-Masip M Dayan P Dolan RJ . 2013. Effort and valuation in the brain: the effects of anticipation and execution. J Neurosci 33:6160–9. 10.1523/JNEUROSCI.4777-12.2013 PMC3639311 23554497

[bibr76-1073858420985447] Kurniawan IT Guitart-Masip M Dolan RJ . 2011. Dopamine and effort-based decision making. Front Neurosci 5:81. 21734862 10.3389/fnins.2011.00081 PMC3122071

[bibr77-1073858420985447] Kurniawan IT Seymour B Talmi D Yoshida W Chater N Dolan RJ . 2010. Choosing to make an effort: the role of striatum in signaling physical effort of a chosen action. J Neurophysiol 104:313–21. 10.1152/jn.00027.2010 PMC2904211 20463204

[bibr78-1073858420985447] Lafargue G Franck N. 2009. Effort awareness and sense of volition in schizophrenia. Conscious Cogn 18:277–89. 10.1016/j.concog.2008.05.004 18653358

[bibr79-1073858420985447] Lafargue G Franck N Sirigu A. 2006. Sense of motor effort in patients with schizophrenia. Cortex 42:711–9. 10.1016/s0010-9452(08)70409-x 16909631

[bibr80-1073858420985447] Lafargue G Paillard J Lamarre Y Sirigu A. 2003. Production and perception of grip force without proprioception: is there a sense of effort in deafferented subjects? Eur J Neurosci 17:2741–9. 10.1046/j.1460-9568.2003.02700.x 12823481

[bibr81-1073858420985447] Lamb F Anderson J Saling M Dewey H. 2013. Predictors of subjective cognitive complaint in postacute older adult stroke patients. Arch Phys Med Rehabil 94:1747–52. 10.1016/j.apmr.2013.02.026 23529143

[bibr82-1073858420985447] Linder J Wenngren BI Stenlund H Forsgren L. 2012. Impaired oculomotor function in a community-based patient population with newly diagnosed idiopathic parkinsonism. J Neurol 259:1206–14. 10.1007/s00415-011-6338-9 22173951

[bibr83-1073858420985447] Luu BL Day BL Cole JD Fitzpatrick RC . 2011. The fusimotor and reafferent origin of the sense of force and weight. J Physiol (Lond) 589:3135–47. 10.1113/jphysiol.2011.208447 PMC3145930 21521756

[bibr84-1073858420985447] Manjaly ZM Harrison NA Critchley HD Do CT Stefanics G Wenderoth N , and others. 2019. Pathophysiological and cognitive mechanisms of fatigue in multiple sclerosis. J Neurol Neurosurg Psychiatry 90:642–51. 10.1136/jnnp-2018-320050 PMC6581095 30683707

[bibr85-1073858420985447] Mead GE Graham C Dorman P Bruins SK Lewis SC Dennis MS , and others. 2011. Fatigue after stroke: baseline predictors and influence on survival. Analysis of data from UK patients recruited in the International Stroke Trial. PLoS One 6:e16988. 21445242 10.1371/journal.pone.0016988 PMC3060800

[bibr86-1073858420985447] Morgante F Dattola V Crupi D Russo M Rizzo V Ghilardi MF , and others. 2011. Is central fatigue in multiple sclerosis a disorder of movement preparation? J Neurol 258:263–72. 10.1007/s00415-010-5742-x 20859746

[bibr87-1073858420985447] Nelson A Schneider DM Takatoh J Sakurai K Wang F Mooney R. 2013. A circuit for motor cortical modulation of auditory cortical activity. J Neurosci 33:14342–53. 10.1523/JNEUROSCI.2275-13.2013 PMC3761045 24005287

[bibr88-1073858420985447] Ormstad H Aass HCD Lund-Sørensen N Amthor KF Sandvik L. 2011. Serum levels of cytokines and C-reactive protein in acute ischemic stroke patients, and their relationship to stroke lateralization, type, and infarct volume. J Neurol 258:677–85. 10.1007/s00415-011-6006-0 PMC3065641 21424610

[bibr89-1073858420985447] Otto T Zijlstra FRH Goebel R. 2014. Neural correlates of mental effort evaluation—involvement of structures related to self-awareness. Soc Cogn Affect Neurosci 9:307–15. 10.1093/scan/nss136 PMC3980803 23202660

[bibr90-1073858420985447] Palotai M Cavallari M Koubiyr I Morales Pinzon A Nazeri A Healy BC , and others. 2019. Microstructural fronto-striatal and temporo-insular alterations are associated with fatigue in patients with multiple sclerosis independent of white matter lesion load and depression. Mult Scler 26:1708–18. 10.1177/1352458519869185 31418637

[bibr91-1073858420985447] Patejdl R Penner IK Noack TK Zettl UK . 2016. Multiple sclerosis and fatigue: a review on the contribution of inflammation and immune-mediated neurodegeneration. Autoimmun Rev 15:210–20. 10.1016/j.autrev.2015.11.005 26589194

[bibr92-1073858420985447] Pauletti C Mannarelli D Locuratolo N Currà A Marinelli L Fattapposta F. 2019. Central fatigue and attentional processing in Parkinson’s disease: an event-related potentials study. Clin Neurophysiol 130:692–700. 30875536 10.1016/j.clinph.2019.01.017

[bibr93-1073858420985447] Peelle JE . 2018. Listening effort: how the cognitive consequences of acoustic challenge are reflected in brain and behavior. Ear Hear 39:204–14. 10.1097/AUD.0000000000000494 PMC5821557 28938250

[bibr94-1073858420985447] Penner IK Paul F. 2017. Fatigue as a symptom or comorbidity of neurological diseases. Nat Rev Neurol 13:662–75. 10.1038/nrneurol.2017.117 29027539

[bibr95-1073858420985447] Perelmuter JT Wilson AB Sisneros JA Forlano PM . 2019. Forebrain dopamine system regulates inner ear auditory sensitivity to socially relevant acoustic signals. Curr Biol 29:2190–2198.e3. 31204161 10.1016/j.cub.2019.05.055

[bibr96-1073858420985447] Petrides M Pandya DN . 2002. Comparative cytoarchitectonic analysis of the human and the macaque ventrolateral prefrontal cortex and corticocortical connection patterns in the monkey. Eur J Neurosci 16:291–310. 12169111 10.1046/j.1460-9568.2001.02090.x

[bibr97-1073858420985447] Philbeck JW Witt JK . 2015. Action-specific influences on perception and postperceptual processes: present controversies and future directions. Psychol Bull 141:1120–44. 10.1037/a0039738 PMC4621785 26501227

[bibr98-1073858420985447] Piitulainen H Bourguignon M Smeds E De Tiège X Jousmäki V Hari R. 2015. Phasic stabilization of motor output after auditory and visual distractors. Hum Brain Mapp 36:5168–82. 10.1002/hbm.23001 PMC6869540 26415889

[bibr99-1073858420985447] Porcaro C Cottone C Cancelli A Rossini PM Zito G Tecchio F. 2019. Cortical neurodynamics changes mediate the efficacy of a personalized neuromodulation against multiple sclerosis fatigue. Sci Rep 9:18213. 31796805 10.1038/s41598-019-54595-z PMC6890667

[bibr100-1073858420985447] Poulsen MB Damgaard B Zerahn B Overgaard K Rasmussen RS . 2015. Modafinil may alleviate poststroke fatigue: a randomized, placebo-controlled, double-blinded trial. Stroke 46:3470–7. 10.1161/STROKEAHA.115.010860 26534969

[bibr101-1073858420985447] Pravatà E Zecca C Sestieri C Caulo M Riccitelli GC Rocca MA , and others. 2016. Hyperconnectivity of the dorsolateral prefrontal cortex following mental effort in multiple sclerosis patients with cognitive fatigue. Mult Scler 22:1665–75. 10.1177/1352458515625806 26846988

[bibr102-1073858420985447] Proske U Allen T. 2019. The neural basis of the senses of effort, force and heaviness. Exp Brain Res 237:589–99. 10.1007/s00221-018-5460-7 30604022

[bibr103-1073858420985447] Reznik D Henkin Y Levy O Mukamel R. 2015a. Perceived loudness of self-generated sounds is differentially modified by expected sound intensity. PLoS One 10:e0127651. 25992603 10.1371/journal.pone.0127651 PMC4436370

[bibr104-1073858420985447] Reznik D Mukamel R. 2019. Motor output, neural states and auditory perception. Neurosci Biobehav Rev 96:116–26. 10.1016/j.neubiorev.2018.10.021 30391407

[bibr105-1073858420985447] Reznik D Ossmy O Mukamel R. 2015b. Enhanced auditory evoked activity to self-generated sounds is mediated by primary and supplementary motor cortices. J Neurosci 35:2173–80. 10.1523/JNEUROSCI.3723-14.2015 PMC6705360 25653372

[bibr106-1073858420985447] Rönnbäck L Hansson E. 2004. On the potential role of glutamate transport in mental fatigue. J Neuroinflammation 1:22. 15527505 10.1186/1742-2094-1-22 PMC533886

[bibr107-1073858420985447] Rummell BP Klee JL Sigurdsson T. 2016. Attenuation of responses to self-generated sounds in auditory cortical neurons. J Neurosci 36:12010–26. 10.1523/JNEUROSCI.1564-16.2016 PMC6604922 27881785

[bibr108-1073858420985447] Salamone JD Yohn SE López-Cruz L Miguel NS Correa M. 2016. Activational and effort-related aspects of motivation: neural mechanisms and implications for psychopathology. Brain 139:1325–47. 10.1093/brain/aww050 PMC5839596 27189581

[bibr109-1073858420985447] Sandroni P Walker C Starr A. 1992. “Fatigue” in patients with multiple sclerosis. Motor pathway conduction and event-related potentials. Arch Neurol 49:517–24. 10.1001/archneur.1992.00530290105019 1580815

[bibr110-1073858420985447] Sanes JN Shadmehr R. 1995. Sense of muscular effort and somesthetic afferent information in humans. Can J Physiol Pharmacol 73:223–33. 10.1139/y95-033 7621361

[bibr111-1073858420985447] Schneider DM Nelson A Mooney R. 2014. A synaptic and circuit basis for corollary discharge in the auditory cortex. Nature 513:189–94. 10.1038/nature13724 PMC4248668 25162524

[bibr112-1073858420985447] Shangyan H Kuiqing L Yumin X Jie C Weixiong L. 2018. Meta-analysis of the efficacy of modafinil versus placebo in the treatment of multiple sclerosis fatigue. Mult Scler Relat Disord 19:85–9. 10.1016/j.msard.2017.10.011 29175676

[bibr113-1073858420985447] Sherman SM . 2017. Functioning of circuits connecting thalamus and cortex. Compr Physiol 7:713–39. 10.1002/cphy.c160032 28333385

[bibr114-1073858420985447] Shetty T Cogsil T Dalal A Kim E Halvorsen K Cummings K , and others. 2019. High-sensitivity C-reactive protein: retrospective study of potential blood biomarker of inflammation in acute mild traumatic brain injury. J Head Trauma Rehabil 34:E28–E36. 30499931 10.1097/HTR.0000000000000450

[bibr115-1073858420985447] Siciliano M Trojano L Santangelo G De Micco R Tedeschi G Tessitore A. 2018. Fatigue in Parkinson’s disease: a systematic review and meta-analysis. Mov Disord 33:1712–23. 10.1002/mds.27461 30264539

[bibr116-1073858420985447] Slobounov S Hallett M Newell Karl M. 2004. Perceived effort in force production as reflected in motor-related cortical potentials. Clin Neurophysiol 115:2391–402. 10.1016/j.clinph.2004.05.021 15351382

[bibr117-1073858420985447] Spiteri S Hassa T Claros-Salinas D Dettmers C Schoenfeld MA . 2019. Neural correlates of effort-dependent and effort-independent cognitive fatigue components in patients with multiple sclerosis. Mult Scler 25:256–66. 10.1177/1352458517743090 29160739

[bibr118-1073858420985447] Stephan KE Manjaly ZM Mathys CD Weber LAE Paliwal S Gard T , and others. 2016. Allostatic self-efficacy: a metacognitive theory of dyshomeostasis-induced fatigue and depression. Front Hum Neurosci 10:550. 27895566 10.3389/fnhum.2016.00550 PMC5108808

[bibr119-1073858420985447] Su SH Xu W Li M Zhang L Wu YF Yu F , and others. 2014. Elevated C-reactive protein levels may be a predictor of persistent unfavourable symptoms in patients with mild traumatic brain injury: a preliminary study. Brain Behav Immun 38:111–7. 10.1016/j.bbi.2014.01.009 24456846

[bibr120-1073858420985447] Takarada Y Mima T Abe M Nakatsuka M Taira M. 2014. Inhibition of the primary motor cortex can alter one’s “sense of effort”: effects of low-frequency rTMS. Neurosci Res 89:54–60. 25264373 10.1016/j.neures.2014.09.005

[bibr121-1073858420985447] Tanaka M Ishii A Watanabe Y. 2013. Neural mechanisms underlying chronic fatigue. Rev Neurosci 24:617–28. 10.1515/revneuro-2013-0035 24114898

[bibr122-1073858420985447] Taylor JL Amann M Duchateau J Meeusen R Rice CL . 2016. Neural contributions to muscle fatigue: from the brain to the muscle and back again. Med Sci Sports Exerc 48:2294–306. 10.1249/MSS.0000000000000923 PMC5033663 27003703

[bibr123-1073858420985447] Tecchio F Cancelli A Cottone C Ferrucci R Vergari M Zito G , and others. 2015. Brain plasticity effects of neuromodulation against multiple sclerosis fatigue. Front Neurol 6:141. 26191036 10.3389/fneur.2015.00141 PMC4490242

[bibr124-1073858420985447] Tecchio F Cancelli A Cottone C Zito G Pasqualetti P Ghazaryan A , and others. 2014. Multiple sclerosis fatigue relief by bilateral somatosensory cortex neuromodulation. J Neurol 261:1552–8. 10.1007/s00415-014-7377-9 24854634

[bibr125-1073858420985447] Tecchio F Zito G Zappasodi F Dell’Acqua ML Landi D Nardo D , and others. 2008. Intra-cortical connectivity in multiple sclerosis: a neurophysiological approach. Brain 131:1783–92. 10.1093/brain/awn087 18502782

[bibr126-1073858420985447] Thickbroom GW Sacco P Kermode AG Archer SA Byrnes ML Guilfoyle A , and others. 2006. Central motor drive and perception of effort during fatigue in multiple sclerosis. J Neurol 253:1048–53. 10.1007/s00415-006-0159-2 16607472

[bibr127-1073858420985447] Tiemann L Heitmann H Schulz E Baumkötter J Ploner M. 2014. Dopamine precursor depletion influences pain affect rather than pain sensation. PLoS One 9:e96167. 24760082 10.1371/journal.pone.0096167 PMC3997524

[bibr128-1073858420985447] Tomasevic L Zito G Pasqualetti P Filippi M Landi D Ghazaryan A , and others. 2013. Cortico-muscular coherence as an index of fatigue in multiple sclerosis. Mult Scler 19:334–43. 10.1177/1352458512452921 22760098

[bibr129-1073858420985447] Uddin LQ Nomi JS Hébert-Seropian B Ghaziri J Boucher O. 2017. Structure and function of the human insula. J Clin Neurophysiol 34:300–6. 10.1097/WNP.0000000000000377 PMC6032992 28644199

[bibr130-1073858420985447] Valdés-Baizabal C Carbajal GV Pérez-González D Malmierca MS . 2020. Dopamine modulates subcortical responses to surprising sounds. PLoS Biol 18:e3000744. 32559190 10.1371/journal.pbio.3000744 PMC7329133

[bibr131-1073858420985447] Visser MM Maréchal B Goodin P Lillicrap TP Garcia-Esperon C Spratt NJ , and others. 2019. Predicting modafinil-treatment response in poststroke fatigue using brain morphometry and functional connectivity. Stroke 50:602–9. 10.1161/STROKEAHA.118.023813 30777001

[bibr132-1073858420985447] Westbrook A Lamichhane B Braver T. 2019. The subjective value of cognitive effort is encoded by a domain-general valuation network. J Neurosci 39:3934–47. 10.1523/JNEUROSCI.3071-18.2019 PMC6520500 30850512

[bibr133-1073858420985447] Whitehead LC Unahi K Burrell B Crowe MT . 2016. The experience of fatigue across long-term conditions: a qualitative meta-synthesis. J Pain Symptom Manage 52:131–143.e1. 27233142 10.1016/j.jpainsymman.2016.02.013

[bibr134-1073858420985447] Willard A Lueck CJ . 2014. Ocular motor disorders. Curr Opin Neurol 27:75–82. 24300789 10.1097/WCO.0000000000000054

[bibr135-1073858420985447] Williamson JW McColl R Mathews D Mitchell JH Raven PB Morgan WP . 2001. Hypnotic manipulation of effort sense during dynamic exercise: cardiovascular responses and brain activation. J Appl Physiol 90:1392–9. 10.1152/jappl.2001.90.4.1392 11247939

[bibr136-1073858420985447] Wu S Duncan F Anderson NH Kuppuswamy A Macloed MR Mead GE . 2015. Exploratory cohort study of associations between serum C-reactive protein and fatigue after stroke. PLoS One 10:e0143784. 26599129 10.1371/journal.pone.0143784 PMC4658028

[bibr137-1073858420985447] Yousif N Fu RZ Abou-El-Ela Bourquin B Bhrugubanda V Schultz SR Seemungal BM . 2016. Dopamine activation preserves visual motion perception despite noise interference of human V5/MT. J Neurosci 36:9303–12. 10.1523/JNEUROSCI.4452-15.2016 PMC5013183 27605607

[bibr138-1073858420985447] Zénon A Sidibé M Olivier E. 2015. Disrupting the supplementary motor area makes physical effort appear less effortful. J Neurosci 35:8737–44. 10.1523/JNEUROSCI.3789-14.2015 PMC6605204 26063908

[bibr139-1073858420985447] Zhang JJ Ding J Li JY Wang M Yuan YS Zhang L , and others. 2017. Abnormal resting-state neural activity and connectivity of fatigue in Parkinson’s disease. CNS Neurosci Ther 23:241–7. 10.1111/cns.12666 PMC6492678 28044431

[bibr140-1073858420985447] Zhou M Liang F Xiong XR Li L Li H Xiao Z , and others. 2014. Scaling down of balanced excitation and inhibition by active behavioral states in auditory cortex. Nat Neurosci 17:841–50. 10.1038/nn.3701 PMC4108079 24747575

